# Genome wide screen identifies microsatellite markers associated with acute adverse effects following radiotherapy in cancer patients

**DOI:** 10.1186/1471-2350-11-123

**Published:** 2010-08-11

**Authors:** Yuichi Michikawa, Tomo Suga, Atsuko Ishikawa, Hideki Hayashi, Akira Oka, Hidetoshi Inoko, Mayumi Iwakawa, Takashi Imai

**Affiliations:** 1 RadGenomics Project, Research Center for Charged Particle Therapy, National Institute of Radiological Sciences, Chiba, Japan; 2 Department of Molecular Life Science and Molecular Medicine, Tokai University School of Medicine, Kanagawa, Japan

## Abstract

**Background:**

The response of normal tissues in cancer patients undergoing radiotherapy varies, possibly due to genetic differences underlying variation in radiosensitivity.

**Methods:**

Cancer patients (n = 360) were selected retrospectively from the RadGenomics project. Adverse effects within 3 months of radiotherapy completion were graded using the National Cancer Institute Common Toxicity Criteria; high grade group were grade 3 or more (n = 180), low grade group were grade 1 or less (n = 180). Pooled genomic DNA (gDNA) (n = 90 from each group) was screened using 23,244 microsatellites. Markers with different inter-group frequencies (Fisher exact test *P *< 0.05) were analyzed using the remaining pooled gDNA. Silencing RNA treatment was performed in cultured normal human skin fibroblasts.

**Results:**

Forty-seven markers had positive association values; including one in the *SEMA3A *promoter region (P = 1.24 × 10^-5^). *SEMA3A *knockdown enhanced radiation resistance.

**Conclusions:**

This study identified 47 putative radiosensitivity markers, and suggested a role for *SEMA3A *in radiosensitivity.

## Background

A principle determinant of the efficiency of tumor eradication following radiotherapy is the total radiation dose given to a patient. The radiation tolerance of important organs located at the margins of the radiotherapy target volume is a critical issue in determining dose thresholds. However, variation in the genetic background of individuals also contributes to the severity of radiation-related adverse events [1-7].

Andreassen *et al. *have summarized the results of many genetic association studies that used single nucleotide polymorphisms (SNPs) as genetic markers, and compared allele frequencies in radiosensitive and nonradiosensitive individuals [7]. Most studies use a candidate gene approach; with genes selected based on ontology. In particular, these studies have focused on genes involved in processes including response to DNA damage, cell death, cell cycle control, oxidative stress, radiation-induced fibrogenesis, and endothelial cell damage.

Systematic microarray gene expression analyses [8-13] and *in vitro *functional screening using siRNA knockdown of gene expression [14], have been used to identify potential radiation susceptibility genes. Significant association has been found between the risk of early adverse skin reactions (EASRs) following radiotherapy, and SNP haplotypes associated with six of 137 candidate genes (*CD44*, *MAD2L2*, *PTTG1*, *RAD9A*, *LIG3 *and *REV3L*) [15]. This has led to the development of a novel DNA chip-based technique to analyze haplotype markers in individual cancer patients [16-19].

Although positive associations between genetic markers and radiosensitivity have been found, the search for strongly associated genetic markers has been unrewarding [7], and this is partly due to inadequate understanding of the molecular pathology of adverse reactions induced by radiotherapy.

Microsatellites are useful mapping tools as they are abundant and interspersed throughout the human genome, similar to SNPs. Importantly though, microsatellite polymorphism generally exceeds that of single SNPs, even reaching the degree of polymorphism provided by SNP haplotypes [20]. Thus, association analyses using a relatively small number of microsatellites should still have adequate statistical power relative to that provided by SNPs [20]. This is illustrated by the identification of genes associated with rheumatoid arthritis [21], narcolepsy [22] and Behcet's disease [23] using genome-wide association studies based on microsatellites.

Hence, a genome-wide association study was performed to identify candidate genes that are strongly associated with radiosensitivity in humans. The screen analyzed data from 23,244 microsatellites in 360 cancer patients who had undergone radiotherapy and been graded for normal tissue adverse reactions. Forty-seven markers were identified as being of interest, with a role for the involvement of *SEMA3A *in radiosensitivity suggested.

## Methods

### Grading of patients with low and high grade radiosensitivity

Since 2001, the RadGenomics project has enrolled more than 3000 patients who have undergone radiotherapy. All patients provided written informed consent to participate in this study, which was approved by the Institutional Review Board at the National Institute of Radiological Sciences and by each collaborating institution. The acute adverse reactions of individual patients up to three months after completion of radiotherapy were graded according to the National Cancer Institute's Common Toxicity Criteria (NCICTC) version 2. We retrospectively selected 180 patients, who presented with a severe acute reaction of equal to or greater than grade 3, as a high-grade group (HGG). We also retrospectively selected 180 patients with less than or equal to grade 1 acute reaction on any end point, as a low-grade group (LGG). The assignment of patients to the LGG and HGG took into consideration their cancer type, age, gender, treatment type, and radiation dose (table 1).

**Table 1:** Clinical features for patients in the genome screen

Characteristic	initial genome screen		focused screen		Difference between groups (P)
			
	LGG-1	HGG-1	LGG-2	HGG-2	
	(n = 90)	(n = 90)	(n = 90)	(n = 90)	
Age at RT					
Mean ± SD	59.7 ± 14.6	59.7 ± 11.5	62.5 ± 12.8	59.5 ± 11.1	0.434
Range	26-89	38-83	38-88	21-79	
					
Family history of cancer	33 (36.7%)	23 (25.6%)	42 (46.7%)	37 (41.1%)	0.129
					
Cancer type					
Breast	42	7	42	7	
Cervical	15	27	15	27	
Esophageal	4	15	4	15	
Head and neck	10	30	9	31	
Lung	18	10	19	9	
Prostate	1	1	1	1	
					
TNM classification					
T1	50	13	46	22	
T2	30	30	27	35	
T3	8	34	11	26	
T4	2	13	6	7	
					
N0	72	43	72	40	
N1	12	30	13	27	
N2	6	10	4	17	
N3	0	6	1	5	
N4	0	0	0	1	
					
M0	90	78	87	81	
M1	0	12	3	9	
					
Dose of radiotherapy (Gy)*					
Breast	49.6 ± 1.4		49.9 ± 0.6		0.602
Cervical	50.2 ± 4.3		50.7 ± 3.9		0.704
Esophageal	54.2 ± 11.3		50.7 ± 8.6		0.776
Head and neck	65.0 ± 5.9		65.3 ± 3.9		0.8
Lung	52.1 ± 8.9		51.4 ± 10.8		0.715
					
Grade of adverse event**					
0	30	0	25	0	
1	60	0	65	0	
2	0	0	0	0	
3	0	84	0	74	
4	0	6	0	16	

* Average dose was obtained only for patients treated with photon therapy. Prostate cancer was treated using brachytherapy or particle therapy.

**Adverse event mainly: dermatitis for breast cancer; diarrhea, bone marrow suppression for cervical cancer; dysphagia, bone marrow suppression for esophageal cancer; dermatitis, mucositis for head and neck cancer; pneumonitis, bone marrow suppression for lung cancer; dysuria for prostate cancer.

Abbreviations: LGG = Low grade group, HGG = High grade group, RT = radiotherapy, TNM = Tumor Node Metastasis.

### Preparation of pooled DNA samples

Extraction of gDNA from whole blood was performed using an automatic nucleic acid isolator, NA3000S (Kurabo, Osaka, Japan) or with the QIAamp DNA blood kit (Qiagen, Hilden, Germany). The gDNA concentrations were measured in triplicate using a PicoGreen double-stranded DNA quantification kit (Invitrogen, Carlsbad, USA) and an SF600 microtiter plate reader (Corona Electric, Ibaraki, Japan). To reduce the amount of genotyping required, gDNA samples were pooled according to the method of Collins *et al *[24]. Concentrations of individual gDNA samples were adjusted to 8 ng/μL. An equal volume of each of 90 gDNA samples from the HGG was combined to generate the first set of pooled gDNA and termed HGG-1. Similarly, 90 gDNA samples from the LGG were pooled and termed LGG-1. A second set of pooled gDNA samples was also prepared from 90 samples of the HGG and 90 samples of the LGG, and these were termed HGG-2 and LGG-2, respectively.

### Analysis of microsatellite markers

All microsatellite markers and the methods for microsatellite analysis used in this study are described in Tamiya *et al *[21]. The genomic location of the microsatellite markers was investigated using the UCSC Genome Browser http://genome.ucsc.edu/cgi-bin/hgGateway. PCR primers to amplify microsatellites were designed to anneal at 57°C, with forward primers having a 5' fluorescent label (6-FAM or HEX). PCR was performed using the GeneAmp PCR system 9700 (GE Healthcare, Amersham Place, UK) in 20 μL containing 48 ng pooled DNA, 0.5 U AmpliTaq DNA polymerase, reaction buffer containing 1.5 mM MgCl_2 _(GE Healthcare, Amersham Place, UK), 5 μM of each primer, and 0.25 mM of each dNTP in 96- or 384-well plates. PCR profile was as follows; 96°C for 5 min, 57°C for 1 min, 72°C for 1 min; 40 cycles of 96°C for 45 s, 57°C for 45 s, 72°C for 1 min. For microsatellite typing of individual samples, PCR was performed in 12 μL containing 2 ng DNA, 0.25 U AmpliTaq Gold DNA polymerase (GE Healthcare, Amersham Place, UK), reaction buffer containing 1.5 mM MgCl_2_, 5 μM of each primer, and 0.2 mM of each dNTP in 96- or 384-well plates and amplified as above. PCR products were denatured in Hi-Di formamide (GE Healthcare, Amersham Place, UK) at 95°C for 3 min and separated by capillary electrophoresis using an ABI Prism 3700 Genetic Analyzer and ROX size standards (GE Healthcare, Amersham Place, UK). Analysis of fragment size and electrophoretograms was performed using GeneScan and Genotyper software (GE Healthcare, Little Chalfont, UK).

### Statistical analysis

Allele frequencies in pooled DNA were estimated from the height of peaks in the electrophoretogram [21]. Association of microsatellites with radiosensitivity was assessed using Fisher exact test and 2 × 2 contingency tables for each allele. The lowest *P *value for any allele of a particular microsatellite was used in analysis for that microsatellite marker and significance was set at 0.05. To account for multiple testing across the microsatellite markers, the *P *values of the second screening were adjusted using the false discovery rate (FDR) controlling procedure of Benjamini and Hochberg [25]. Association between particular alleles of a microsatellite and grade of radiosensitivity was performed using the Cochran-Armitage test for trend [26].

### Cell culture conditions

Normal human skin fibroblast NB1RGB cells were obtained from Riken Cell Bank (Tsukuba, Japan) and maintained in Eagle's minimum essential medium (Nissui, Tokyo, Japan) supplemented with 10% fetal bovine serum (FBS) and nonessential amino acids under a humidified atmosphere of 5% CO_2 _at 37°C.

### siRNA treatment of human fibroblast cultures

Two different siRNAs (1: 135598, 2: 135597, Applied Biosystems/Ambion, Austin, USA) designed for the human *SEMA3A *gene were used to treat cells by reverse transfection. Cultured cells were harvested by incubation with 0.05% trypsin, 0.53 mM EDTA in phosphate-buffered saline (trypsin-EDTA/PBS) for 5 min at room temperature, followed by inactivation of the trypsin by adding complete culture medium. The number of cells with a diameter of 12 μm was measured using the Z1 Coulter Particle Counter (Beckman Coulter, Brea, USA). Transfection complexes were prepared in 266.6 μl Opti-MEM serum free medium by mixing 5.4 μL of siPORT NeoFX Transfection Reagent (Applied Biosystems/Ambion, Austin, USA) for 10 min prior to adding 2.7 μl of 10 μM siRNA (Applied Biosystems/Ambion, Austin, USA) for 10 min. The cell suspension containing 27,000 cells in 725.3 μl was added to the transfection complexes and the mixture plated onto a 35 mm plastic dish. Cells were maintained under a humidified atmosphere of 5% CO_2 _at 37°C for 24 hours. The mock control cells were treated similarly, except the transfection complexes were prepared without siRNA. The transfection medium was then changed to complete culture medium, and cells were maintained for the indicated time.

### Western blotting

The post-transfection complete culture medium was recovered at the indicated time, centrifuged at 12,000 × g for 5 min and the supernatant transferred to a microfuge tube and stored at -30°C until use. Fifteen μL aliquots of supernatant were mixed with 5 μL of loading dye, incubated at 80°C for 10 min and loaded onto NuPAGE 4-12% polyacrylamide Bis-Tris gradient gels (Invitrogen, Carlsbad, USA) under denaturing conditions. Proteins in the gels were then transferred onto PVDF membranes by the iBlot Gel Transfer Device (Invitrogen, Carlsbad, USA). The membranes were probed with primary rabbit polyclonal antibody SEMA (H-300) against amino acids 103-402 of the human protein from *SEMA3A *(Santa Cruz Biotechnology, Santa Cruz, USA). A secondary donkey anti-rabbit IgG conjugated to horseradish peroxidase (GE Healthcare, Amersham Place, UK) was used to detect protein bands using an ECL Advance Western Blotting Detection Kit (GE Healthcare, Little Chalfont, UK).

### Radiosensitivity assay of siRNA-treated human skin fibroblast cultures

Cells cultured for 48 hours following replacement of the siRNA transfection medium, were harvested by incubation with trypsin-EDTA/PBS for 5 min at room temperature. Trypsin was then inactivated by adding complete culture medium, and the number of cells with a diameter of more than 12 μm was counted using a Z1 Coulter Particle Counter. The appropriate number of cells (1,000 cells for 0 Gy, 1,500 cells for 1 Gy, 3,000 cells for 2 Gy, 6,000 cells for 3 Gy and 12,000 cells for 4 Gy) were then irradiated at room temperature with 200 kV X-rays (20 mA) with 0.5 mm aluminum and 0.5 mm copper filters. Immediately after irradiation, cells were plated onto 100 mm (0 and 1 Gy) or 150 mm (2-4 Gy) plastic dishes, and cultured under a humidified atmosphere of 5% CO_2 _at 37°C for 2 weeks. Cells were washed with PBS, then fixed in 100% methanol for a few minutes at room temperature. After removing the methanol, the cells were dried for 30 minutes and then stained with 3% Giemsa solution for 2 hours. Colonies consisting of more than 50 cells were scored as survivors. Experiments were performed with triplicate plating of cells. Relative colony survival as a function of irradiated dose was tested using the linear-quadratic model [27].

## Results

### Genome-wide association study

**Figure 1:**
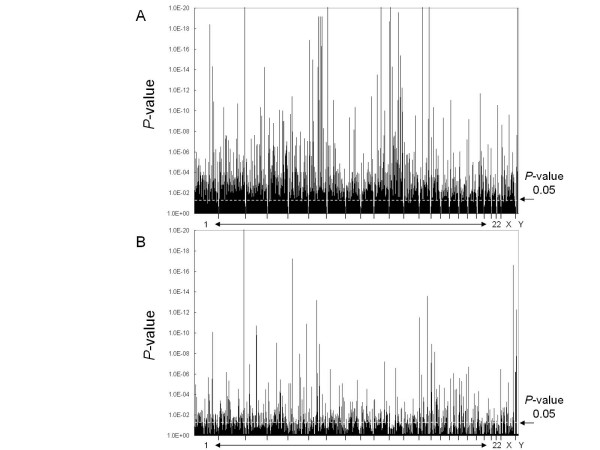
**Results of two rounds of screening of microsatellite markers for association with radiosensitivity**. (A) Results of the screen of the entire genome using 23,244 microsatellite markers and the HGG-1 and LGG-1 pooled DNAs. (B) Results of screening 3,052 markers of interest identified in the preceding genome scan and using the HGG-2 and LGG-2 pooled DNAs. *P *values calculated using Fisher's exact test and 2 × 2 contingency tables are shown. The level of significance (*P *< 0.05) is shown as a horizontal broken line.

The entire human genome was screened using 23,244 microsatellite markers and one set of the pooled DNA from each group (HGG-1 and LGG-1). This analysis identified 3,052 markers with allele/s that showed significantly different estimated frequency (*P *< 0.05) between the two groups (see figure 1A). These markers were further analyzed in the second round screening with the remaining set of pooled DNA samples from each group (HGG-2 and LGG-2). A total of 101 markers had allele/s with significantly different estimated frequency (*P *< 0.05) between the two groups, and had similar peak association patterns in the data from the first and second DNA pools (see figure 1B). The FDR of the markers analyzed in the second round screening was then estimated to correct for the effect of multiple comparisons. After correction for the multiple comparisons performed, 47 autosomal markers had a FDR < 0.05, and so were significantly associated with radiosensitivity in this study. These markers are summarized in table 2.

**Table 2:** List of markers showing positive association with radiosensitivity

Number	Marker	Chr	Position in hg18*		Sequence of PCR primers		*P *value		FDR
						
			5'-end	3'-end	Forward	Reverse	1st	2nd	
1	D5S0329i	5	33286356	33286817	GAAAGAATTACACTTTGCCAA	AATTGAGATGTCTAAAGGCATC	2.549E-05	5.916E-18	3.897E-15
2	D6S0876i	6	82039209	82039634	ACCACAATAAGATATCACCTCAC	CATTACATTCATGCATACACATAC	0.0263	6.518E-14	1.717E-11
3	D3S0313i	3	104474152	104474509	AACCATCAGATCTTGTTAGAATTA	GTAGTGGTACAGGCCTGTAGT	2.409E-07	2.082E-11	3.048E-09
4	D1S0494i	1	199551754	199551974	GTGTAAGAGCCAGCTGGAC	TCTTGTTTGTGTCTGGTATTAGG	0.0066	8.736E-11	1.151E-08
5	D3S0923i	3	105397288	105397735	GGTATCATTTATCTGGATCCAC	AATTCTTTCAGATGAAAGGAAG	4.025E-04	1.714E-10	2.052E-08
6	D5S0365i	5	111127178	111127405	ACACAAATAAGCATGCGC	CCAGATTATCCTACCACGC	1.127E-08	1.127E-08	9.279E-07
7	D14S0444i	14	26355444	26355548	TCATAGCACTTATCACTACCTTAGG	GATGAAGCCAAGGAGGAG	3.863E-08	8.37E-08	5.80E-06
8	D7S0192i	7	24587185	24587633	ATTACCATTATGGTAGGCTGAATA	GCCAGACATTTGAAATCAGTAT	0.0025	3.53E-07	1.95E-05
9	D5S0292i	5	117035880	117036332	ACTTATAGGTCAGCAACCATTC	GCAATTAGATGGCATTAAATTA	2.82E-04	2.27E-06	8.80E-05
10	D17S945	17	9763962	9764159	AACCAATCTGGACTCCCC	CCTGAAGCCTGACCCC	4.52E-04	2.70E-06	1.02E-04
11	D4S0813i	4	122056656	122057077	TCTTCAGCATTCAGAATATGAT	CAAATTGTCTTTGTTATGTACCTC	4.98E-04	3.66E-06	1.27E-04
12	D8S0809i	8	121379855	121380093	AAGTTATTTATACCAAGTGATGGTG	TCCTAGGATCCTAAGATATAAATCTG	0.0455	3.89E-06	1.32E-04
13	D5S1310i	5	2137543	2137692	AGCCTTCACTCGTGTTCTTAC	AAATAGGAGGACAGAGTAGCAGAG	4.72E-04	8.25E-06	2.41E-04
14	D7S1017i	7	135196215	135196636	ACTGTTCTCATGGTAGTGAATAAG	CTCTTCCTCGTTTGTAGACAC	0.0039	8.78E-06	2.52E-04
15	D9S0704i	9	82887428	82887533	TGCCATGACTGTCTTTCC	TGGAACAAATGATATCAAGAGATAG	0.006	3.02E-05	6.22E-04
16	D7S0072i	7	147781781	147782230	TTCATGAATCCTCAACTATTAAAG	GCTTACTACCTGGTTAATGAAATA	0.0081	2.11E-04	0.0030
17	D20S0325i	20	47715303	47715449	GTTATGATCATGTCACTGCAC	CCTTAGGACTTGATGTTTCTTC	0.0069	2.39E-04	0.0034
18	D10S0385i	10	7092531	7092986	GGATAACTCCAAGATTTCTGAC	GATTAACATGTAAATAGGCAGACT	0.0143	2.90E-04	0.0038
19	D8S0335i	8	116305649	116306128	TTAATATTGATAACATCTTGCGA	CCTTATGACAAATCTTTTCTGAG	0.0266	4.40E-04	0.0053
20	D3S0046i	3	6944093	6944453	ATGGCCATGTTATGATGTTAAT	TGACAGAGCTAGACTCTTGTCAG	0.0247	4.98E-04	0.0054
21	D14S0356i	14	75942526	75942991	TACTCAAATGTCACATTGGTTA	CAGACTAATCAATAACAGAGGACT	8.30E-04	5.00E-04	0.0054
22	D15S966	15	96766304	96766555	TGCTGCTCACGAACTTTT	CCTCTTGGGAACTGTGTAGTATT	0.0142	4.87E-04	0.0054
23	D9S0416i	9	7107471	7107725	AATATTACCTTATTGTTGAGTAAATGC	CCAACAATATAATTTAGGAAGAGC	0.0187	0.0014	0.0127
24	D13S0138i	13	100138248	100138637	AGACTCATCTCCTACCTTTCAC	TAAGAATCCATATGTTGCTGAC	0.0017	0.0017	0.0147
25	D9S0242i	9	8735688	8736162	CGTTTGTAATTGAACGAATAAC	CACCTTGTCACAATTACTCAAC	0.0087	0.0019	0.0160
26	HUMUT6930B	21	39672158	39672587	CGGAGGTTGCAGTGAGTTG	GGGAAGGCTATGGAGGAGA	0.0007	0.0022	0.0174
27	D8S0912i	8	89632493	89632925	GGATGTCTTGTCTTACATTCTCTAG	CAGGTGTCTACTAAACCATCTGAC	0.0231	0.0023	0.0178
28	D6S0444i	6	9065374	9065718	AATTAAGAGTGATCTGAGCAGAAG	TCAAATTTGGTGACCTTAACAG	0.0016	0.0023	0.0182
29	D5S0803i	5	23507111	23507556	TGGTATTAATCTGTTAATGAGGAC	GAATCAACCTAAGTGTACATCAAC	0.0357	0.0024	0.0186
30	D18S0429i	18	487260	487643	CGGATCACCATATAGTGAAAC	GAATTAGAAAGTCACGTTGGTAC	0.0269	0.0026	0.0194
31	HUMUT544	11	123087682	123087978	ACTTCAGCCTCGGTGACAG	TGTTCTGCCTCTGTTGTTAC	0.0453	0.0027	0.0202
32	D4S0317i	4	64967890	64968058	GATACAATTGGTACTGCAAAATAC	TCCTAAGTTCCTAAAGAATCACTC	0.0000	0.0030	0.0215
33	D8S0674i	8	60955922	60956375	TTTCTAATCCATCTGTCAATAAAG	AGTCACTATGCATAGACCACAC	0.0004	0.0031	0.0220
34	D7S2422	7	51105545	51105739	GCTCCACATTCCTTGGGTA	AAGTGAGGGCCTTTCAAAC	0.0131	0.0037	0.0249
35	D10S0463i	10	73857334	73857571	AGAGTAAATCCTCTCCTCATACAAG	TTGCACAGTTCAGAAGCC	0.0046	0.0045	0.0288
36	D8S0225i	8	30387109	30387478	CAGTTGGAATAAATCAGTGAAC	CTGGCTAACATGGTGAAAC	0.0077	0.0070	0.0370
37	D10S0809i	10	48104477	48104600	GCTAGTGTTGGGAGTCAGC	AGGTGAGCCAGTTTCCAG	0.0123	0.0068	0.0370
38	D20S0115i	20	36321601	36321804	ATACTGGCACAAACGCATC	TTAGACTCAGCTATGGAAAGTCAG	0.0013	0.0068	0.0370
39	D12S0456i	12	64866094	64866556	ATTTACCTATGTAACAAACTGCAC	CACAGACTTAATGGATAAGCAG	0.0042	0.0075	0.0383
40	D4S0132i	4	30434753	30434922	CCAGAAGATAGAAGGAGAATCAC	TCTTGATGGTTGGCTGTC	0.0300	0.0085	0.0401
41	D7S0338i	7	83663532	83663831	CTTAACAATACTGGCTGATCAATAG	CATTGTCACTACAGCTTTCATTAC	0.0013	0.0088	0.0413
42	D12S0341i	12	33909083	33909516	TCAAACCTTCATCTTTGTACTTAG	AACTGGCTTCTATCTCACCTAT	0.0223	0.0089	0.0416
43	D1S0288i	1	33214510	33214921	ATACTGTACTTCATGCCATAAGAC	GAGAAATACGGCTGGTGTA	0.0000	0.0092	0.0425
44	D1S1215i	1	241929347	241929516	CTGCAGTCACAACTGGTTAAG	TTGATAACAGCATACTTCAACATAG	0.0027	0.0097	0.0430
45	D12S1657	12	96183565	96183714	TCCTAAAGATGGTGTGCAT	AAGTTCCAATGTTAGTGAACC	0.0431	0.0100	0.0435
46	D16S0497i	16	59449366	59449516	GTAAATCCAATATCTTGCCTACAG	TTCAGGTTCTCCATGTCAAC	0.0361	0.0104	0.0446
47	D10S0107i	10	119415495	119415711	ACAGGCAATCTGATAGTTTAAGAG	AATTGACTCAAGGTTCTGCAG	0.0254	0.0112	0.0469

* hg18: human genome assembly version 18

Abbreviation: Chr = Chromosome,

### Individual patient typing of selected markers

The genomic location of the 47 positive markers was determined using the UCSC Genome Browser. Four markers were within 15 kb upstream of the transcription start site for the nearest gene (table 3). To confirm the associations observed in the pooled DNA analysis, these four markers were individually typed in all 360 patients. The particular alleles of D1S0288i and D7S0338i found to be statistically significantly associated with radiosensitivity in the experiments using pooled DNA, were also found to be so when experiments using individual DNA samples were used (table 3). One of the positive markers, D7S0338i, was selected for further study as it had the strongest association with radiosensitivity.

**Table 3:** Reproducibility of markers located within 15 kb distance to the nearest transcription start sit

Chr	Marker	Repeat Unit	Nearest gene	Distance to TS	**2**^**nd **^**screen FDR**	*P *values		
						
						**1**^**st **^**screen**	**2**^**nd **^**screen**	Overall
1	D1S0288i	GAA	*IBRDC3*	12196 bp	0.043	0.019	0.041	0.001
			(IBR domain					
			containing 3)					
								
5	D5S0365i	TG	*C5orf13*	6571 bp	9.28E-07	0.031	0.639	0.054
			(Protein p311)					
								
7	D7S0338i	GA	*SEMA3A*	1453 bp	0.041	0.012	2.70E-04	1.24E-05
			(Semaphorin-3A)					
								
12	D12S0456i	TG	*IRAK3*	2796 bp	0.038	0.018	0.194	0.008
			(Interleukin-1 receptor-					
			associated kinase 3)					

Abbreviation: Chr = chromosome, TS = transcription start site, FDR = false discovery rate

### Detailed association analysis of the D7S0338i marker

Allelic distribution of the marker D7S0338i is represented in additional file 1. Eleven alleles were identified in D7S0338i, with three major alleles (292 bp, 294 bp and 296 bp) accounting for more than 90% of the alleles in the study population (see table 4 and additional file 1). Among them, two alleles (292 bp and 296 bp) had an overall *P *value < 0.05. Association analysis revealed the 292/292 genotype was associated with risk reduction (odds ratio: 0.490, 95% CI: 0.271-0.885), the 294/294 genotype was associated with intermediate risk (odds ratio: 0.794, 95% CI: 0.321-1.966), and the 296/296 genotype was associated with an increased risk (odds ratio: 2.861, 95% CI: 1.417-5.774) (see table 5). Interestingly, the heterozygote individuals carrying the risk reducing and risk increasing alleles (292/296) had intermediate risk (odds ratio: 1.152, 95% CI: 0.727-1.824). This suggests the inter-allele relationship may not behave in the usual dominant-recessive manner. A linear trend was identified between six combinations of alleles for the marker and radiosensitivity (*P *= 7.228 × 10^-4^) (see table 5).

**Table 4:** Allelic association analysis for the D7S0338i marker using Fisher's exact test

	**1**^**st **^**screen**			**2**^**nd **^**screen**			Overall results		
			
Allele (bp)	LGG n(%)	HGG n(%)	*P *value	LGG n(%)	HGG n(%)	*P *value	LGG n(%)	HGG n(%)	*P *value
278	0 (0.0)	1 (0.6)	1.000	1 (0.6)	1 (0.6)	1.000	1 (0.3)	2 (0.6)	1.000
282	0 (0.0)	0 (0.0)	1.000	1 (0.6)	1 (0.6)	1.000	1 (0.3)	1 (0.3)	1.000
284	1 (0.6)	0 (0.0)	0.494	0 (0.0)	0 (0.0)	1.000	1 (0.3)	0 (0.0)	0.496
288	1 (0.6)	1 (0.6)	1.000	0 (0.0)	1 (0.6)	1.000	1(0.3)	2 (0.6)	1.000
290	0 (0.0)	1 (0.6)	1.000	0 (0.0)	0 (0.0)	1.000	0 (0.0)	1 (0.3)	1.000
292	88 (50.0)	68 (37.8)	0.025	79 (44.4)	58 (32.2)	0.022	167 (47.2)	126 (35.0)	0.001
294	37 (21.0)	39 (21.7)	0.898	45 (25.3)	37 (20.6)	0.315	82 (23.2)	76 (21.1)	0.529
296	44 (25.0)	68 (37.8)	0.012	47 (26.4)	81 (45.0)	2.70E-04	91 (25.7)	149 (41.4)	1.24E-05
298	4 (2.3)	1 (0.6)	0.211	4 (2.2)	1 (0.6)	0.214	8 (2.3)	2 (0.6)	0.062
300	0 (0.0)	1 (0.6)	1.000	0 (0.0)	0 (0.0)	1.000	0 (0.0)	1 (0.3)	1.000
302	1 (0.6)	0 (0.0)	0.494	1 (0.6)	0 (0.0)	0.497	2 (0.6)	0 (0.0)	0.245

**Table 5:** Genotype association analysis of D7S0338i marker

Number	Genotype	LGG	HGG	*P *value	Odds ratio	95% CI
						
		n (%)	n (%)			
1*	292/292	36 (20.3)	20 (11.1)	0.017	0.490	0.271-0.885
2*	292/294	41 (23.2)	29 (16.1)	0.093	0.637	0.375-1.081
3*	294/294	11 (6.2)	9 (5.0)	0.788	0.794	0.321-1.966
4*	292/296	48 (27.1)	54 (30.0)	0.547	1.152	0.727-1.824
5*	294/296	17 (9.6)	28 (15.6)	0.090	1.734	0.912-3.295
6*	296/296	12 (6.8)	31 (17.2)	0.002	2.861	1.417-5.774
7	Others	12 (6.8)	9 (5.0)	0.624	0.724	0.297-1.763

*Cochran-Armitage trend test: *P *= 7.228 × 10^-4^

### Impact on cellular radiosensitivity of the D7S0338i associated gene SEMA3A

**Figure 2:**
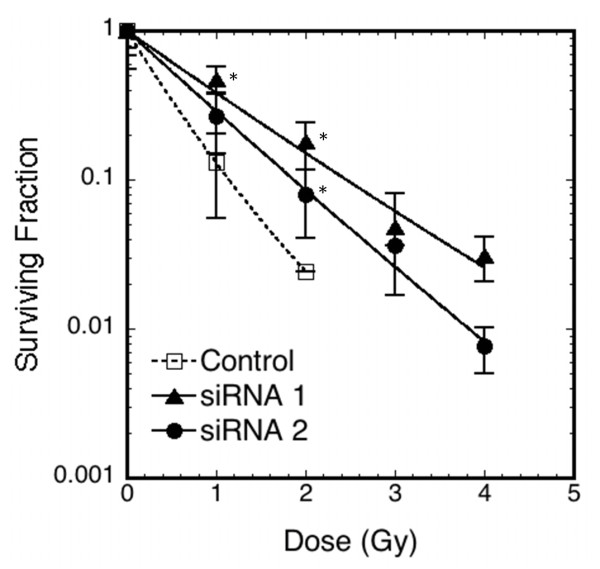
***In vitro *radiosensitivity assay on human skin fibroblast cells treated with *SEMA3A *gene-directed siRNA**. The relative clonogenic survival rate of cells at the indicated X-ray dose (Gy) is plotted. Open box: mock control. Closed triangle: *SEMA3A *siRNA1. Closed circle: *SEMA3A *siRNA2. The linear-quadratic model [27] of cell survival as a function of irradiated dose was fitted to the data. The asterisk indicates significant difference between the siRNA transfected cells and the control cells (*P *value < 0.05).

The D7S0338i marker is 1500 bp upstream of the transcription start site of the *SEMA3A *gene (table 3). The linkage disequilibrium block around this marker did not extend to neighbouring genes (see additional file 2). To assess possible functional impacts of the *SEMA3A *gene on cellular radiosensitivity (see additional file 3), a model system using *in vitro *irradiation of cultured fibroblasts derived from human skin was employed. Specific *SEMA3A *siRNA treatment of these cells reduced expression of the *SEMA3A *gene in normal human skin fibroblasts (see additional file 4). When these cells were irradiated with X-rays (200 keV), the colony survival assay of the irradiated cells showed enhancement of cellular radiation resistance in the *SEMA3A *siRNA-treated cells (see figure 2).

## Discussion

A human genome-wide microsatellite association study was performed in cancer patients who showed radiation-induced adverse reactions. After correction for multiple comparisons, this study identified 47 autosomal markers with a FDR < 0.05. One of these markers is within the proximal promoter region of the *SEMA3A *gene on chromosome 7. Knockdown of *SEMA3A *expression in a normal human skin fibroblast culture caused a significant change in the radiosensitivity of these cells.

The *SEMA3A *gene has not been previously described as having a role in radiosensitivity. *SEMA3A *encodes a secreted protein (semaphorin-3A), which is involved in a wide range of functional processes including regulation of axon guidance, cell survival, motility, immune responses and angiogenesis [28-49] (see additional file 3). This diversity of these roles provides many possible mechanisms for its involvement in radiosensitivity. Semaphorin-3A is also a competitor of the angiogenic growth factor coded for by the *VEGF *gene, as both bind to the same transmembrane receptor [29,31,46]. As *VEGF *expression is directly correlated with radiosensitivity [50], its competitor, semaphorin-3A, may also be associated with radiosensitivity.

The potential role of the D7S0338i marker in radiosensitivity is interesting. The microsatellite is located 1500 bp upstream from the transcription start site of *SEMA3A*, and is a GA dinucleotide repeat (see additional file 5). Two other polymorphic repetitive sequences also occur between the D7S0338i marker and the *SEMA3A *transcription start site (additional file 6). These three repetitive sequences are in a low nucleosome occupancy region (data not shown). Since nucleosomes play a major role in generating the higher order structure of chromatin that regulates gene expression [51], these sequences may affect the activity of the *SEMA3A *promoter. A study into the activity of the *SEMA3A *promoter may provide information on the functional impacts of the D7S0338i marker polymorphism, especially mechanisms underlying the phenotypes associated with various alleles (table 5).

A major limitation of association studies on rare phenotypes such as the severe (equal to or greater than grade 3), acute, adverse reactions induced by radiotherapy, is the ability to enroll sufficient numbers of patients to provide adequate statistical power [7,52]. The reproducibility of any association identified must also be replicated [53], further increasing the required patient number. Hence in this study, cancer patients were selected with differing severe, acute, adverse reaction endpoints and various cancer types. Identical numbers of control patients were selected who did not develop severe, adverse reactions on any endpoint. The clinical characteristics and therapeutic protocols used in the control and subject patients were also similar (table 1). While this suggests inherent or genetic differences between patients are the cause of the variations in severity in patient reactions, the involvement of *SEMA3A *requires further validation using large numbers of patients with a unique cancer type.

A possible implication for this study's finding that semaphorin-3A may be involved in radiosensitivity, is the identification of a potential new agent for the treatment of radiotherapy-induced damage. SM-216289 (xanthofulvin) was originally isolated from the fermentation broth of a fungal strain, *Penicillium sp*. SPF-3059, and is a natural inhibitor of Semaphorin-3A [54]. SM-216289 abolished the growth cone collapse of dorsal root ganglion neurons that was induced by Semaphorin-3A *in vitro *and *in vivo*, possibly through direct interference of the receptor-ligand association [54]. Local administration of SM-216289 in the adult rat model of spinal cord injury, substantially enhanced functional recovery of injured axons, with decreases in apoptotic cell number and marked enhancement of angiogenesis [55]. Therefore, locally administered SM-216289 may aid functional recovery in radiotherapy-induced injury.

Increasingly, phenotypic differences have been shown to be caused by diverse genetic variations. While SNP and repetitive DNA polymorphisms have been shown to be disease-associated, other disease-associated changes include copy number variation [56] and transgenerational epigenetic modification of the genome [57]. Thus, diagnostic testing should be considered to identify highly radiosensitive cancer patients through the detection of genetic variants in individual patients. The rapidly developing next-generation genome sequencing technology [58] may be the most suitable one for this purpose.

## Conclusions

A total of 47 putative markers for individual radiosensitivity were identified using a genome-wide screen based on microsatellite markers. One of these markers is in the proximal promoter region of *SEMA3A*, with knockdown of this gene using siRNA supporting its potential role in radiosensitivity.

## Competing interests

The authors declare that they have no competing interests.

## Authors' contributions

YM participated in study conception and design, conducted experiments and statistical analyses, participated in data interpretation, and drafted the manuscript. TS conducted experiments and statistical analysis. AI conducted statistical analyses and participated in data interpretation. HH, AO and HI conducted statistical analyses, participated in data interpretation, and revised the article critically for important intellectual content. MI and TI participated in study conception and design, conducted statistical analyses, participated in data interpretation, and revised the article critically for important intellectual content. All authors read and approved the final manuscript

## Pre-publication history

The pre-publication history for this paper can be accessed here:

http://www.biomedcentral.com/1471-2350/11/123/prepub

## Supplementary Material

Additional file 1 **Allele frequencies for the D7S0338i marker**. (A) First screening samples. (B) Second screening samples. (C) Overall results. The number of patients with the indicated allele size is plotted. Reproducible allele frequency differences were observed between the groups. White bar: LGG. Black bar: HGG. Significant differences *(P *< 0.05 by Fisher's exact test based on 2 × 2 contingency tables) are indicated by the vertical lines. Click here for file

Additional file 2 **Linkage disequilibrium map around the D7S0338i marker**. Diagram showing the region of linkage disequilibrium (D') surrounding the *SEMA3A *gene and neighbouring genes. An increase in the degree of linkage disequilibrium between the *SEMA3A *gene and its neighbours is represented by an increase in the intensity of red color. Thus, the D7S0338i marker is in the linkage disequilibrium block that spans the transcriptional regulatory region of the *SEMA3A *gene. The D' data was calculated from JPT and CHB HapMap data. Positions are NCBI build 36 coordinates. Gene region and direction of transcription are indicated by an arrow. Click here for file

Additional file 3 References on semaphorin-3A functions Click here for file

Additional file 4 **Reduction in the amount of semaphorin-3A protein following siRNA treatment**. The amount of semaphorin-3A in the culture medium of cells treated by the siRNA was quantified by Western blotting. The protein amounts relative to that of mock control cells are plotted. Click here for file

Additional file 5 **Sequence of the D7S0338i marker**. This marker is the GA dinucleotide repeat sequence indicated by the red color. The primer sequences for PCR amplification are indicated by the arrows. Click here for file

Additional file 6 **Location of the D7S0338i marker in the promoter region of the *SEMA3A *gene**. The GA dinucleotide repeat sequence is indicated by an open box. Two other polymorphic repetitive sequences (closed box) exist in the region between the D7S0338i marker and the transcription start site (+1). Click here for file
