# Supplementary figures and images for: Genome wide screen identifies microsatellite markers associated with acute adverse effects following radiotherapy in cancer patients
Source: BMC Med Genet. 2010 Aug 11;11:123. doi: 10.1186/1471-2350-11-123 (PMC2928773; doi:10.1186/1471-2350-11-123)

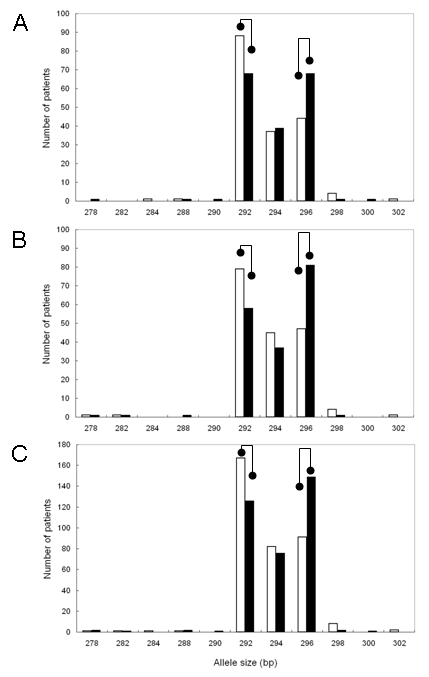

Supplement: Additional file 1 — Allele frequencies for the D7S0338i marker. (A) First screening samples. (B) Second screening samples. (C) Overall results. The number of patients with the indicated allele size is plotted. Reproducible allele frequency differences were observed between the groups. White bar: LGG. Black bar: HGG. Significant differences (P < 0.05 by Fisher's exact test based on 2 × 2 contingency tables) are indicated by the vertical lines. [file 1471-2350-11-123-S1.TIFF]

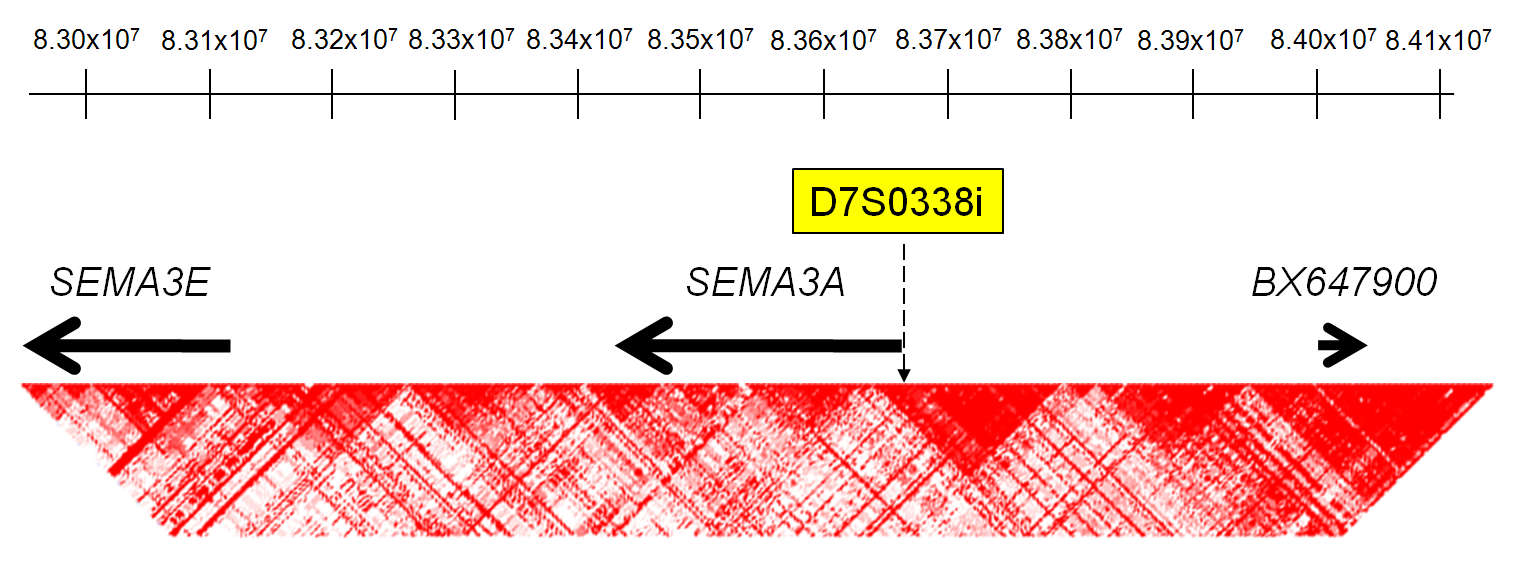

Supplement: Additional file 2 — Linkage disequilibrium map around the D7S0338i marker. Diagram showing the region of linkage disequilibrium (D') surrounding the SEMA3A gene and neighbouring genes. An increase in the degree of linkage disequilibrium between the SEMA3A gene and its neighbours is represented by an increase in the intensity of red color. Thus, the D7S0338i marker is in the linkage disequilibrium block that spans the transcriptional regulatory region of the SEMA3A gene. The D' data was calculated from JPT and CHB HapMap data. Positions are NCBI build 36 coordinates. Gene region and direction of transcription are indicated by an arrow. [file 1471-2350-11-123-S2.TIFF]

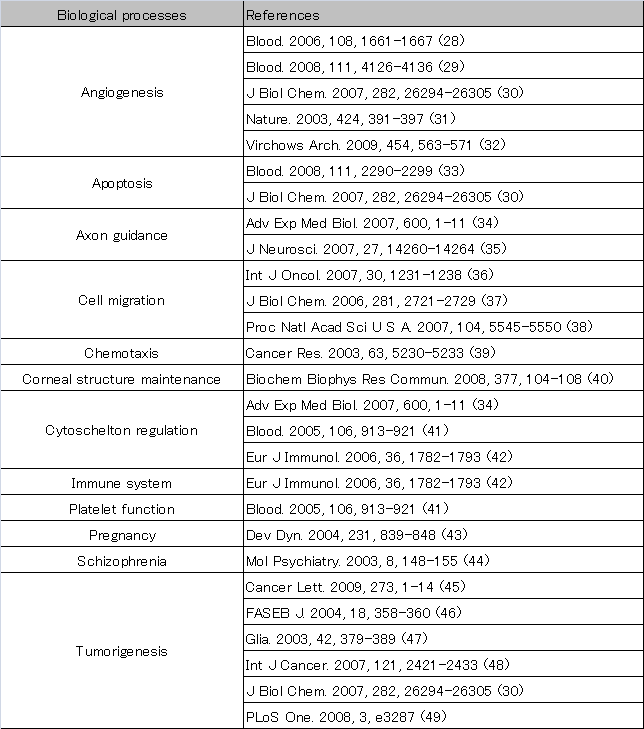

Supplement: Additional file 3 — References on semaphorin-3A functions [file 1471-2350-11-123-S3.TIFF]

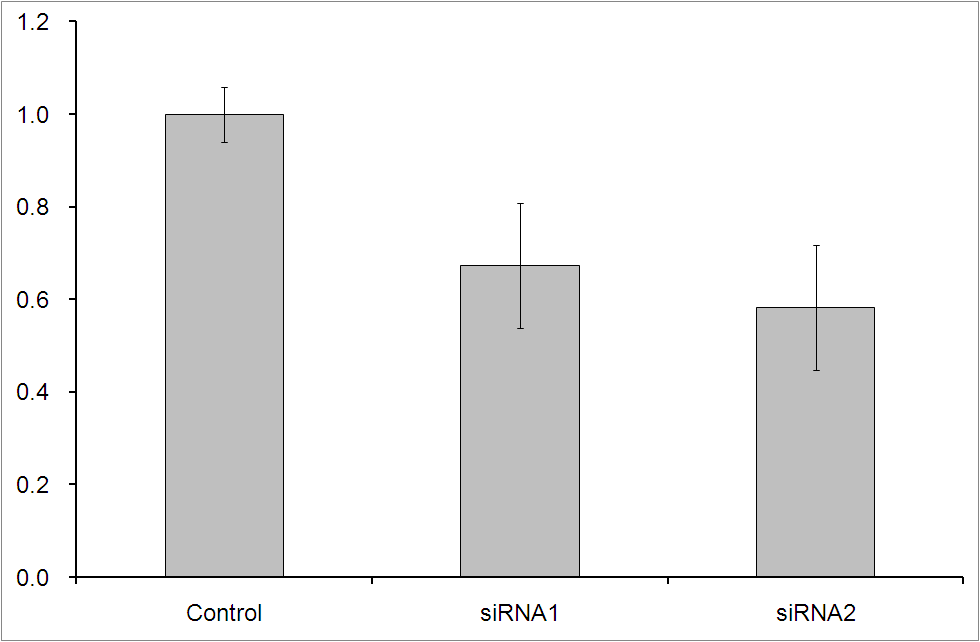

Supplement: Additional file 4 — Reduction in the amount of semaphorin-3A protein following siRNA treatment. The amount of semaphorin-3A in the culture medium of cells treated by the siRNA was quantified by Western blotting. The protein amounts relative to that of mock control cells are plotted. [file 1471-2350-11-123-S4.TIFF]

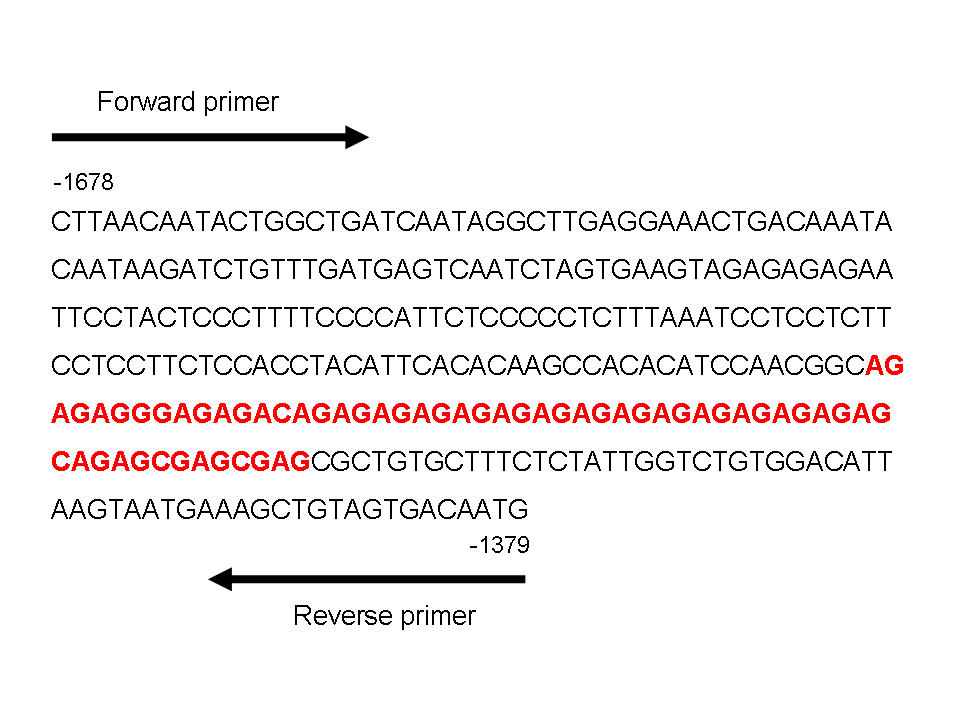

Supplement: Additional file 5 — Sequence of the D7S0338i marker. This marker is the GA dinucleotide repeat sequence indicated by the red color. The primer sequences for PCR amplification are indicated by the arrows. [file 1471-2350-11-123-S5.TIFF]

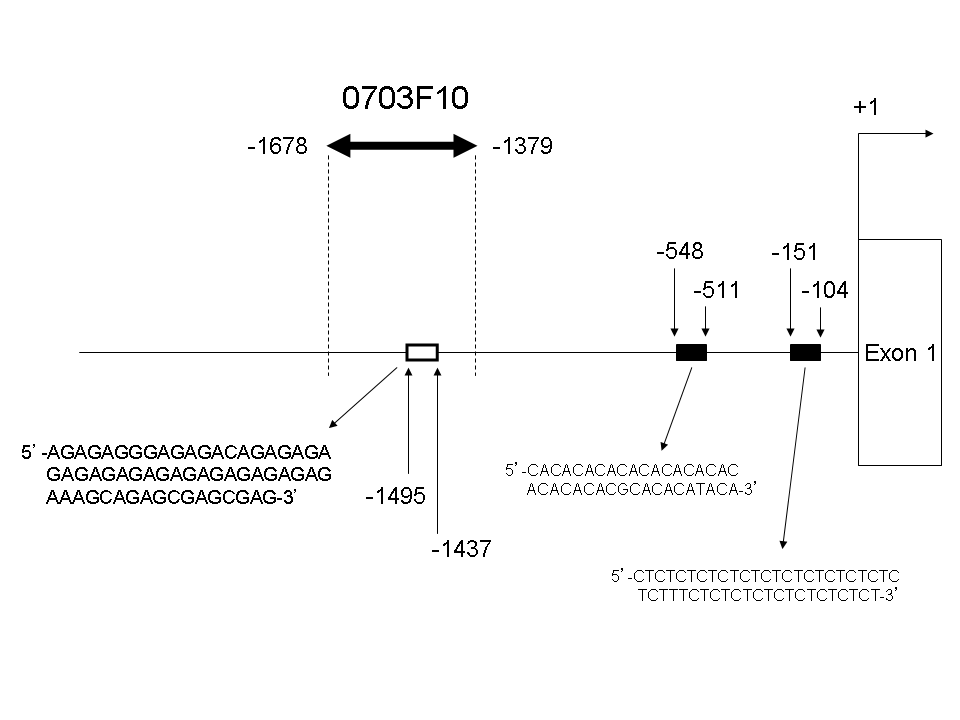

Supplement: Additional file 6 — Location of the D7S0338i marker in the promoter region of the SEMA3A gene. The GA dinucleotide repeat sequence is indicated by an open box. Two other polymorphic repetitive sequences (closed box) exist in the region between the D7S0338i marker and the transcription start site (+1). [file 1471-2350-11-123-S6.TIFF]
